# An Infrastructure Framework for Remote Patient Monitoring Interventions and Research

**DOI:** 10.2196/51234

**Published:** 2024-05-30

**Authors:** Jennifer Claggett, Stacie Petter, Amol Joshi, Todd Ponzio, Eric Kirkendall

**Affiliations:** 1 School of Business Wake Forest University Winston-Salem, NC United States; 2 Center for Healthcare Innovation School of Medicine Wake Forest University Winston-Salem, NC United States; 3 Health Science Center University of Tennessee Memphis, TN United States

**Keywords:** remote patient monitoring, eHealth, telehealth, telemonitoring, telemedicine, digital infrastructure, clinical decision-making

## Abstract

Remote patient monitoring (RPM) enables clinicians to maintain and adjust their patients’ plan of care by using remotely gathered data, such as vital signs, to proactively make medical decisions about a patient’s care. RPM interventions have been touted as a means to improve patient care and well-being while reducing costs and resource needs within the health care ecosystem. However, multiple interworking components must be successfully implemented for an RPM intervention to yield the desired outcomes, and the design and key driver of each component can vary depending on the medical context. This viewpoint and perspective paper presents a 4-component RPM infrastructure framework based on a synthesis of existing literature and practice related to RPM. Specifically, these components are identified and considered: (1) data collection, (2) data transmission and storage, (3) data analysis, and (4) information presentation. Interaction points to consider between components include transmission, interoperability, accessibility, workflow integration, and transparency. Within each of the 4 components, questions affecting research and practice emerge that can affect the outcomes of RPM interventions. This framework provides a holistic perspective of the technologies involved in RPM interventions and how these core elements interact to provide an appropriate infrastructure for deploying RPM in health systems. Further, it provides a common vocabulary to compare and contrast RPM solutions across health contexts and may stimulate new research and intervention opportunities.

## Introduction

### Overview

Remote patient monitoring (RPM; sometimes referred to as eHealth, telehealth, telemonitoring, or telemedicine) involves the capture of patient data through sensors or devices outside of a clinical setting, such as at the patient’s home or work while the patient is engaging in everyday activities. Ideally, the data captured through RPM devices are analyzed and used to inform clinicians’ decisions on patient care. For example, typical decisions include adjusting the recommended dosage or timing of a patient’s medication based on observed changes in the patient’s vital signs or patterns of activity.

RPM interventions have increased exponentially in the United States of America since 2020 [1]. The COVID-19 pandemic exacerbated the need for remote patient care solutions when there were severe resource shortages of clinicians, equipment, and capacity within health care systems [2-4] and patients were required to socially distance themselves to mitigate the spread of COVID-19. As the United States of America eased regulations and made changes to encourage reimbursements for RPM interventions, health care providers sought to reap RPM’s potential benefits along three main dimensions: (1) enhancing quality by offering more personalized care; (2) achieving scale by growing their customer (patient) base; and (3) securing new reimbursement opportunities by evolving in response to shifts in payment policies [1,3].

The excitement and promise of the benefits of RPM to improve patient care while also expanding a health system’s market are well-documented in meta-analyses that find evidence of RPM reducing hospital admissions and length of stay for certain conditions, such as cardiovascular disease or chronic obstructive pulmonary disease [5,6]. Decreased travel time, cost savings, and increased access to services are commonly ascribed as benefits for patients, and most eHealth interventions are described as successes [7]. However, other scholars counter that RPM interventions may not live up to the hype. One study finds that RPM interventions do not impact patient health factors, such as weight, body fat percentage, and blood pressure [8], and other related studies raise concerns about the limited evidence that RPM interventions can indeed adequately scale to meaningfully improve patient outcomes and demonstrably reduce health care costs [3].

These mixed results regarding the impact of RPM interventions showcase the current challenge of understanding how to design and effectively implement the infrastructure to support successful RPM programs. Successful RPM programs should meet at least one, but ideally both, of the following standards: (1) improved management of symptoms (evaluated using population-normalized values or patient feedback) and (2) reduced financial costs (evaluated in terms of the health system, payers, and patient out-of-pocket expenses). Previous work reporting on RPM interventions tends to report details on isolated projects and is focused, understandably, on a specific medical condition without offering generalizable advice to a broader audience or a catalog of best practices. Although RPM has been implemented in many different types of contexts, we contend that the key infrastructure points are consistent across interventions. Therefore, we present a framework consisting of 4 core infrastructure components necessary for any RPM intervention and identify common questions across contexts that should influence the RPM intervention design and results. This RPM infrastructure framework is useful to scholars and clinicians implementing RPM projects in that it (1) presents a shared vocabulary and reference point, (2) serves as a resource to guide some of the major decisions associated with an RPM implementation, and (3) provides a logical scaffolding to categorize and disseminate lessons learned within RPM projects to leverage them in other contexts. While the set of considerations nested within the four infrastructure components is not exhaustive, these considerations serve as a useful starting point as RPM research and interventions are planned and developed in the future.

### RPM Infrastructure Framework

**Figure 1:**
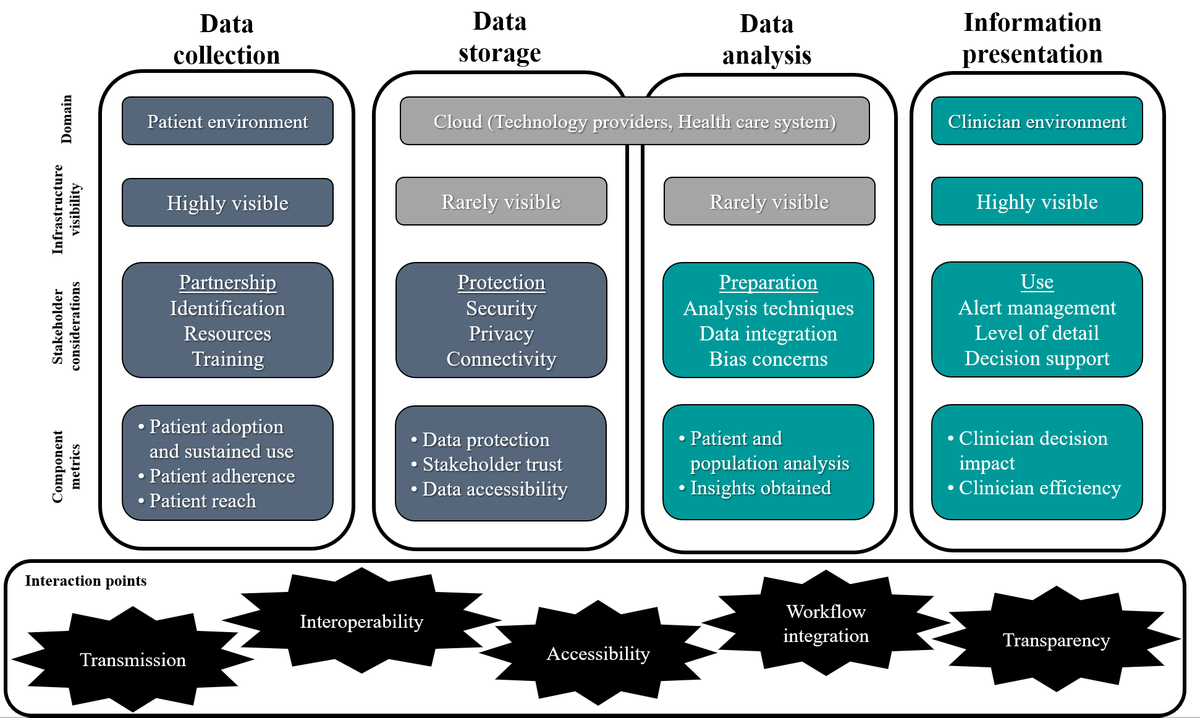
Remote patient monitoring (RPM) infrastructure framework.

As an information technology (IT), RPM relies on a combined and layered infrastructure of hardware, software, and networks to support the collection, storage, processing, and management of data. By considering emergent patterns and themes from the literature, cases, and reports, discussing this topic in various panels and workshops, and reflecting on our experiences designing and assessing RPM projects, we propose a four-component infrastructure framework that is necessary in any RPM infrastructure project: (1) data collection, (2) data transmission and storage, (3) algorithmic data analysis, and (4) information presentation. The first RPM infrastructure component, data collection, collects a patient’s vital signs and other biometric data remotely through a measurement device such as a wearable sensor. Data transmission and storage, the second infrastructure component, leverages software interface services, networking, and hardware to transfer the data from the patient’s device to a centralized data architecture [9,10]. Third, software-based algorithms analyze the stored remote patient data to identify patterns and outliers for a single patient or for a patient population. The final RPM infrastructure component is to present information obtained from the analysis to support clinicians’ decision-making processes [11,12]. Figure 1 depicts the RPM infrastructure framework, and each of the following sections describes key considerations for each component.

## Component 1: Remote Patient Data Collection

### Overview

Patients interact with an RPM device to enable the collection of data outside of clinical settings. Some devices are worn continuously throughout a person’s day, while other devices are used at specific times to capture health indicators periodically based on the patient’s medical condition and the provider’s care protocol. Patients may use a specialized RPM device that registers a single form of biometric data (eg, a continuous glucose monitor capturing blood glucose levels) or a device that captures multiple data types (eg, a blood pressure cuff that measures blood pressure, pulse rate, and oxygen saturation). Given the growing number of technologies capable of collecting patient health data along with the need for patients to interact with a device for data collection, several questions must be carefully addressed when considering how to best collect data for an RPM intervention.

### How Should Patients be Selected for RPM?

While RPM has the potential to improve patients’ quality of care and reduce clinic costs, successful implementation relies on the effective use of the device and the fidelity of the collected data. The existing literature highlights several key considerations and components for identifying patients who are a good match for remote monitoring. Of paramount importance is suitability—is the patient’s medical condition one that is likely to actually benefit from the collection and analysis of more data? Patients with chronic diseases such as diabetes, heart failure, hypertension, or chronic obstructive pulmonary disease are often more likely to benefit from RPM, as it can help them better manage their health status and condition over the long-term [13]. Comorbidities also play a significant role in patient selection, as those with multiple chronic conditions or complex health situations might require more comprehensive monitoring [14]. RPM can provide a more holistic view of their health, making it a potentially valuable tool for these patients; however, the complexity of their medical conditions may limit their ability to adhere to the monitoring program and necessitate more immediate and direct medical interventions.

Patients who are noncompliant or have a history of difficulty adhering to their treatment plans might benefit from RPM, as it can help improve compliance and provide additional support [15]. RPM solutions may make a patient feel more engaged, empowered, and informed through messaging systems that interact with patients on a routinely structured basis [16,17]. Patient motivation and engagement are key factors, as patients who are motivated and engaged in managing their health are more likely to actively participate in and adhere to the RPM program [18].

Other patient-specific factors—commonly referred to as the social determinants of health—such as socioeconomic status, age, and social support should be considered when designing RPM interventions [19]. For instance, patients with lower socioeconomic status might benefit from RPM the most, as it can help reduce health care disparities and provide better access to care [20-22]. A disproportionally large number of people affected by chronic conditions are from socioeconomically disadvantaged groups [23]. Communities of color, immigrants, and women are particularly likely to be in distress from undiagnosed chronic diseases, and even when diagnosed, these populations are more likely than their counterparts to face structural and logistical obstacles to obtaining the appropriate level of intermittent care. So long as they have reliable connectivity to the internet, patients who live in remote or rural areas or have limited access to transportation might benefit from RPM, as it can help overcome geographical barriers to care [18,24]. Age can also play a role in identifying suitable patients for RPM, in that elderly patients or those with age-related conditions may benefit from RPM. The patient’s living situation is another important factor. A strong support system, such as family or caregivers, can facilitate device use, data collection, and overall engagement, making these patients more suitable for RPM [25].

Finally, technological competence plays a crucial role in a patient’s ability to engage with an RPM device. Patients with some level of technology literacy (eg, “digital natives”) are more likely to engage with and effectively use RPM devices and systems [26]. However, patients with lower socioeconomic status or those who are elderly may have lower levels of technological competence or may have other barriers that could limit the effectiveness of an RPM program [27-29]. There is a natural continuum of sophistication and familiarity with devices and the inevitable troubleshooting they often require, and a more “set and forget” approach may be advisable for certain populations.

### Which Device and Which Types of Data?

A fundamental characteristic of RPM is the acquisition of data outside of conventional clinical environments. Consequently, patient data must be collected remotely using sensors and equipment such as wearable devices, mobile phones, or portable devices installed at a patient’s residence or other environments [30]. One strategy involves using data from off-the-shelf, general-purpose smart health consumer electronics purchased by the patient, while another option is to rely on data from specialized devices or software prescribed or supplied by the health care provider. Technological advancements enable the collection, through devices within the RPM infrastructure, of various types of data, such as electrocardiograms, electroencephalograms, heartbeats and respiration rates, oxygen saturation in the blood or pulse oximetry, nervous system signals, blood pressure, body or skin temperature, blood glucose levels, patient weight, and sleep patterns, among others [31].

A crucial consideration is the optimal combination of metrics to be collected for a specific patient. The US Centers for Disease Control report that 51.8% of US adults have at least one chronic condition, and 27.2% have multiple chronic conditions such as obesity, diabetes, and cardiovascular disease [32]. Emerging evidence indicates that RPM initiatives are more likely to succeed when multiple metrics are evaluated concurrently [33]. For instance, compiling data from various physiological sensors measuring heart rate, blood oxygen saturation, and blood glucose levels simultaneously can offer a more comprehensive overview of a patient’s health, which is particularly significant for patients with comorbidities and additional complications. This suggests that the diagnostic value of data can be enhanced by carefully considering what health indicators are needed to manage a patient’s care.

### How Frequently are Data Collected?

Determining the optimal frequency of data collection in RPM scenarios is a critical consideration, as it can significantly impact the effectiveness of patient care and the efficient use of health care resources. The appropriate frequency for data collection depends on various factors, including the severity and type of the patient’s condition, the objectives of monitoring, and the required patient involvement in data collection [34]. For instance, some conditions may necessitate multiple data readings per day, while others may only require weekly monitoring [35]. Passive data collection methods, such as continuous monitoring of vital signs using wearable sensors, can be advantageous for patients requiring frequent monitoring, whereas active data collection methods, which involve patient involvement and interaction, may be suitable for other conditions [16,36]. Passive methods are usually less likely to cause patient burnout and abandonment [37].

Health care providers should consider adopting several best practices to ensure that patients remain engaged and compliant with RPM protocols. These typically include providing personalized and clear instructions, offering training and support to ensure device functionality, improving patients’ understanding and comfort with the technology, and fostering regular remote communication between patients and health care providers [38]. Furthermore, involving patients in the decision-making process regarding their monitoring plans and adjusting the frequency and type of data collection based on their individual needs and preferences can lead to increased patient engagement and satisfaction [13,39].

## Component 2: Remote Patient Data Transmission and Storage

### Overview

Once remote patient data are collected by one or more devices, the data must be transmitted and shared with clinicians, and stored in a data architecture. The manner in which the data are transmitted from an RPM device is dependent on the device and the network access of the patient. RPM data transmission may occur through a network using a wired link, or high-speed wireless link with or without human intervention. In some cases, patients or caregivers may be asked to record readings or values from devices into an app available on their smartphone or computer that will transmit data to the medical provider. Another option could be that a patient must physically visit a clinician’s office with the device to upload the data to the patient’s electronic medical record. The storage of remote patient data may be in a system that is managed by the device manufacturer and accessed through a web portal, and the data may or may not be integrated within the patient’s electronic medical record.

### Is There Sufficient Connectivity?

Connectivity plays a vital role in the successful implementation of RPM, as it enables the transmission of patient data from monitoring devices to health care providers and fosters timely interventions and informed decision-making. Addressing the digital divide is crucial to ensuring equitable access to RPM services, as patients with limited internet access or low digital literacy may face barriers to fully benefiting from RPM [40]. This disparity is particularly concerning for patients from socioeconomically disadvantaged backgrounds, who may experience greater difficulties in accessing health care services and could benefit the most from RPM [40,41]. Some patients may have access to home internet solutions through local internet service providers that include Wi-Fi networks at home, while others may be limited to cellular network access through mobile devices. Often, the latter is subject to slower connections and data caps that place constraints on the patient’s connectivity.

Strategies for addressing connectivity for RPM interventions should consider alternatives, such as the constant connectivity approach or using batch or episodic data uploads when data connections are available [42]. Constant connectivity can facilitate real-time monitoring and immediate interventions, which may be especially beneficial for patients with critical or rapidly changing health conditions [43]. However, this approach may not be feasible for patients living in areas with limited or unreliable internet access or for those who cannot afford consistent connectivity. In these cases, episodic data uploads when a connection is possible may provide a more accessible and cost-effective solution, allowing health care providers to track patient progress and identify potential issues while accommodating the patients’ connectivity limitations [44]. Additionally, some RPM hardware solutions may include direct cellular network connectivity, where the device sends the data through a connection provided by the wearable device to the provider, bypassing the need for a patient home network. These solutions will incur additional costs related to data transmission and may not naturally provide a patient dashboard or a way for patients to easily view data that may traditionally be housed in a patient application.

### Is the Transmission Secure?

The sensitive nature of medical data necessitates robust protection measures to maintain patient privacy and prevent unauthorized access. Data breaches and cyberattacks can have severe consequences for patients and their health care providers, including identity theft, financial loss, and reputational damage [45]. The increasing connectivity of medical devices and the use of cloud-based data storage have created new opportunities for cybercriminals, leading to the emergence of threats such as medjacking [46]. Medjacking, a term coined from “medical device hijacking,” refers to the unauthorized access and manipulation of medical devices, such as pacemakers or insulin pumps, to cause harm to patients or extract sensitive data [47]. As RPM technologies rely on a variety of connected devices for data collection across multiple networks, they can be vulnerable to medjacking and other cybersecurity risks. Furthermore, the rapid expansion of the internet of things in health care has amplified these risks, as a larger number of interconnected devices create more potential entry points for attackers [48,49].

Health care providers and technology developers should prioritize the implementation of robust security measures to mitigate the risks associated with medjacking and other security threats in RPM. These may include strong encryption protocols for data transmission (“in flight”) and storage (“at rest”), regular security updates, and the development of secure communication channels between devices and health care providers [45,48]. Additionally, health care organizations should adopt a proactive approach to security by conducting regular risk assessments, promoting cybersecurity awareness and training among staff, and fostering a culture of security-mindedness [50].

### Can Data Move Across Health Systems Software?

Interoperability is a crucial aspect of RPM projects, as it enables seamless communication and data sharing among different health information systems, devices, and providers. This encompasses not only the technical aspects of data exchange but also the semantic understanding and interpretation of shared data, ensuring that the information can be effectively used by health care providers, patients, and other stakeholders. Effective interoperability contributes to improved patient care by ensuring that clinicians have access to comprehensive and up-to-date medical information, allowing for better decision-making and coordination of care [51]. However, achieving interoperability in RPM poses several challenges, including the need to balance data accessibility with patient privacy and maintain control over personal health information.

One of the primary challenges in achieving interoperability in RPM is the heterogeneity of health information systems and devices used by health care providers. These systems often rely on different (often proprietary) data formats, communication protocols, and standards, which can create barriers to effective information exchange. To address this issue, several major standards have been developed to facilitate interoperability in health IT (eg, [52,53]). For example, the US Department of Health and Human Services Office of the National Coordinator for Health IT released the third version (V3) of the US Core Data for Interoperability in 2022 [54].

Another challenge in achieving interoperability is protecting patient privacy while sharing data freely among authorized health care providers [44]. Using privacy-preserving techniques, such as pseudonymization, which replaces personally identifiable information with unique identifiers to maintain patient anonymity, may reinforce privacy during the transmission of data between systems. However, these approaches must be rigorously tested to systematically mitigate privacy risks [55]. One-way hashing of sensitive identifiers is another technique that can reduce the risk of leakage of personal health identifiers. Additionally, the implementation of access control mechanisms can help ensure that only authorized users can access and share patient data, further safeguarding privacy [56].

A related issue to moving data across health systems is determining the appropriate granularity to share between stakeholders and systems. For example, in a remote blood pressure monitoring project, should each reading be recorded, transmitted, and made available, including any relevant metadata about time, place, and cuff placement, or should only summary data about daily or weekly averages be shared between systems? Like any sensor-based technology, the amount of raw data generated by RPM initiatives may be overwhelming [57]; however, providing only summarized data limits the transparency and future uses of the data.

## Component 3: Algorithmic Analysis of Remote Patient Data

### Overview

Remote patient data that are stored within an information system and are not analyzed provide no value to the patient or the clinician. After transmitting and storing RPM data, they should be processed and analyzed to identify and summarize patterns and trends in individual patients and patient populations [58]. The process of analyzing raw data to deliver actionable insights could also form the basis for financial reimbursement, which is fundamental to any sustainable RPM program.

### What Analysis Techniques are Appropriate?

Data analysis involves the use of algorithms, or a series of steps, to process the data in a meaningful way. Algorithms may use static rule logic, which can be used to draw attention to results over a certain threshold, or they may leverage machine learning techniques to dynamically adapt and learn from large sets of patient data, such as adjusting the threshold based on similar patients with similar conditions recorded in the data [59]. The distinction between static and dynamic rules has implications that need to be explored.

Static rules can be based on established medical guidelines, such as thresholds for vital signs or other clinical parameters, which can help health care providers identify potential health issues and take appropriate actions [60]. While this method can be effective in some cases, it may not account for the unique characteristics and complexities of individual patients, which may limit its ability to provide personalized care [61].

Alternatively, machine learning techniques offer more advanced and adaptable solutions for analyzing RPM data [62]. These techniques use algorithms that can learn from data patterns and make predictions or decisions without being explicitly programmed [63,64]. Machine learning can be used to identify trends, anomalies, and correlations in patient data, enabling health care providers to make more informed decisions and deliver personalized care [65,66]. Adaptive interpretation techniques take RPM data analysis a step further by dynamically adjusting their approach based on real-time patient data. These methods, which often rely on artificial intelligence and machine learning algorithms, can continuously refine their analysis and predictions to better understand the evolving health status of individual patients [63]. This adaptive approach can help health care providers identify subtle changes in patients’ conditions that may not be evident through traditional analysis techniques, leading to more proactive and personalized care [67].

### Which Comorbidities Should be Included in the Analysis?

This question centers around the appropriate complexity level of analyses of RPM solutions. Incorporating comorbidities into the analysis of RPM data can help health care providers better understand the complex interactions between various conditions and their impact on patients’ health. This, in turn, can lead to more accurate and personalized treatment recommendations. Static rules that solely focus on a single condition, such as high blood pressure, may not adequately account for the impact of comorbidities on patients’ overall health status. For instance, a patient with both diabetes and hypertension may require a different treatment approach than a patient with hypertension alone, which is why any given individual should be managed holistically with a consolidated approach, rather than divided by symptoms and specialty [68].

This comprehensive monitoring can provide a more accurate representation of the patient’s health status, allowing health care providers to make more informed decisions regarding treatment and care management [69,70]. However, these solutions may be so patient-specific that cognitive efficiencies and the ability to scale the solution are compromised in the absence of built-in coordination systems with well-defined decision-making heuristics and robust care protocols.

### What Biases Exist Within the Analysis and How Should They be Mitigated?

Biases in the analysis of remote patient data can have a significant impact on the accuracy and effectiveness of health care services. Particularly in machine learning-based analysis techniques, biases can arise from various sources, such as data sampling, measurement errors, or algorithmic design, leading to potentially biased predictions or recommendations [71,72]. It is essential to detect and account for biases to ensure that the solutions provided are equitable and reliable for all patients.

One primary source of bias in data analysis is the data itself. If the training data used to develop machine learning models do not accurately represent the diverse patient population, the resulting models may be skewed toward specific subgroups, leading to suboptimal or even harmful recommendations for other groups [73,74]. For instance, if a model is trained predominantly on data from patients of a particular age, gender, or ethnicity, it may not perform well on patients from other demographics. To mitigate such biases, it is crucial to ensure that the training data are representative of the target patient population, considering factors such as age, gender, ethnicity, and socioeconomic status [75].

Another source of bias can arise from the choice of features or variables used in the analysis. If certain relevant variables are not included, or if irrelevant variables are considered, the resulting predictions or recommendations may be biased or even spurious [76]. Careful feature selection, based on domain knowledge and a thorough understanding of the underlying data, can help address this issue.

Algorithmic biases can also emerge from the choice of machine learning methods or algorithms, as well as their specific implementations. To address this, it is essential to evaluate and compare multiple algorithms and implementations to identify potential biases and select the most appropriate method for the specific application [77]. Patients themselves can serve as their own baselines too, particularly for measurements that do not lend themselves as easily to a population approach (eg, mood and gastric motility).

Lastly, ongoing monitoring and evaluation of the performance of data analysis solutions, including machine learning models, is critical to detecting and addressing biases. Regular assessments of model performance, particularly with respect to various subgroups within the patient population, can help identify potential biases and ensure that the solutions remain equitable and effective for all patients [78].

## Component 4: Presentation of RPM Data to a Clinician

### Overview

Once the data have been analyzed, the results need to be presented as information to support clinicians’ decision-making. Unless the RPM data are used to inform patient care, the RPM intervention will not yield the intended results. Therefore, it is critical that the information is presented in a manner that is likely to inform clinicians as they make decisions that affect specific patients and patient populations.

### Is RPM Information Accessible in the Right Electronic Health Record Software?

Physicians and other clinical decision makers often face significant time constraints and high cognitive workloads in their daily practice, making it challenging for them to manage and monitor patient data effectively. A study by Sinsky et al [79] found that primary care physicians spent nearly half of their workday interacting with EHR systems, leaving them with limited time for direct patient care. The high volume of clinical tasks and responsibilities can lead to cognitive overload, increasing the risk of burnout and negatively impacting the quality of care provided [80]. Given these constraints, it is critical to ensure that RPM data are easily accessible within the existing EHR systems without requiring clinicians to log into additional platforms or apps. Integrating RPM data into EHRs can help streamline clinical workflows and reduce the cognitive burden on health care providers, enabling them to focus on essential tasks such as patient evaluation, diagnosis, and treatment planning [81]. This underscores the importance of seamless integration and interoperability between RPM solutions and EHR systems, ultimately supporting more efficient and effective patient care by easing the pathway of the information being used in decision-making.

One of the key benefits of integrating RPM data into EHR systems is the ability to provide a comprehensive and up-to-date view of a patient’s health status. By combining RPM data with other health information such as medical history, laboratory results, and imaging studies, clinicians can gain a more holistic understanding of a patient’s condition, enabling them to make more informed decisions about treatment plans and care management strategies [82].

Integration of RPM data into EHR systems can also support the development and implementation of clinical decision support (CDS) tools, which can help health care providers make more informed, evidence-based decisions about patient care [83]. By leveraging RPM data, CDS tools can provide real-time alerts or recommendations to clinicians, assisting them in diagnosing, treating, or managing a patient’s condition more effectively.

### How Should the Decision Maker Receive Information?

In the context of RPM solutions, there is a delicate balance between providing exception reporting and summary data reporting. Exception reporting involves the generation of alerts or notifications only when specific events or abnormal values are detected, which require immediate attention from health care providers. This yields the advantage of focusing health care providers’ attention on situations that need prompt intervention, potentially improving the efficiency and timeliness of care and reducing the number of alerts [84]. However, exception reporting may not always provide sufficient context or information about a patient’s overall health status, making it difficult for clinicians to assess the impact of treatment strategies or identify more subtle changes in condition over time. On the other hand, summary data reporting provides a broader overview of a patient’s progress over time, allowing clinicians to evaluate trends and assess the overall effectiveness of treatment plans. Both approaches have their merits and challenges, making the choice between them a critical consideration in RPM projects.

Alert fatigue is a critical concern in the context of RPM solutions, as it can have significant implications for the effectiveness of the system and the quality of patient care. Alert fatigue occurs when health care providers are exposed to a high volume of alerts, leading to desensitization and potentially reduced responsiveness to these notifications [85-87]. This phenomenon has been observed in various clinical settings, including electronic health record systems and CDS tools, where excessive alerts can contribute to cognitive overload, increased stress, and the risk of overlooking critical information [88].

In RPM systems, balancing the type and frequency of messaging is essential to minimize alert fatigue. The choice between push and pull messaging strategies can play a significant role in this regard. Push messaging involves automatically sending alerts or notifications to health care providers, whereas pull messaging requires providers to actively request or retrieve the information. Although push messaging can ensure timely delivery of critical information, it may also contribute to alert fatigue if used indiscriminately or too frequently. Solutions to alleviate this tension may involve tailoring alert thresholds based on individual patient needs, incorporating CDS algorithms to filter and prioritize alerts, and using a combination of push and pull messaging to strike the right balance between proactively notifying providers and allowing them to access information on demand.

### What is the Right Amount of Information to Provide to Decision Makers?

Balancing transparency and detail in the presentation of RPM data with cognitive ease is crucial for ensuring that health care providers effectively use the information in their decision-making processes. While transparency is essential for building trust and understanding of the underlying data analysis, providing excessive detail can overwhelm clinicians and hinder their ability to quickly assimilate the information [89]. Consequently, it is vital to strike an optimal balance between presenting comprehensive information and ensuring cognitive ease for end users.

One approach to achieving this balance is to use a tiered or “drill-down” presentation of data, which allows health care providers to access additional layers of detail only if they require it [90]. This design can present a high-level summary of the patient’s condition and only flag critical alerts, while enabling providers to delve deeper into the data if they desire further context or clarification. This, in turn, helps mitigate information overload and supports more efficient decision-making by prioritizing the most relevant and actionable insights [91]. Moreover, incorporating the principles of cognitive ergonomics and human-centered design can further enhance the usability of RPM solutions. This may involve the use of visual aids, such as graphs, charts, and color-coding, to facilitate rapid comprehension of complex data and even presenting proposed treatment plans based on the algorithmic analysis of the patient’s full record [92] and providing reference statistics from the health system’s relevant patient population.

### Conclusion

The mixed results with RPM interventions have raised concerns about the scalability and value of this technology. This viewpoint paper highlights some of the key questions and core considerations that affect the various infrastructure components of an RPM intervention. Differences between health conditions, metrics, devices, storage, analysis, and information presentation across RPM implementations result in countless permutations. If scholars fail to document and clearly explain the RPM infrastructure and choices made for an RPM implementation, it will be difficult to create an evidence-based research tradition. Having a shared vocabulary and more consistent documentation of the RPM infrastructure can support future literature reviews and meta-analyses seeking to evaluate the outcomes of RPM interventions. The RPM infrastructure framework presented in this article offers scholars a means to describe the different choices and constraints associated with their RPM interventions.

We also identify how each of the infrastructure components can stimulate new research and intervention opportunities in Table 1. While not exhaustive, the list offers a sampling of the many research questions that could be studied to further increase the understanding associated with RPM interventions. The RPM framework offers scholars and clinicians a more comprehensive guide to exploring various aspects of RPM implementation. As a result, they can further optimize the design and functionality of RPM solutions for improved patient care and health care provider experiences.

**Table 1:** Key questions and considerations per remote patient monitoring (RPM) infrastructure component.

Infrastructure component and relevant questions		Research considerations	Intervention considerations
**Data collection**			
	Which patients?	Examining lesser-studied chronic and acute health conditions that could benefit from RPM interventions Exploring which comorbidities benefit from RPM data collection Determining ways to improve patient compliance with RPM protocols Understanding how to identify and improve technology competence among patients suitable for RPM interventions	Using RPM interventions for health conditions with known positive patient outcomes Collecting additional data through RPM based on the comorbidities of the patient Identifying patient populations most likely to benefit from RPM intervention outcomes Developing strategies to increase technology competence among patient populations who can benefit from RPM interventions
	Which device and data?	Comparing the effectiveness of device types (eg, invasive vs noninvasive and prescribed devices vs consumer electronics) for RPM Identifying which metrics (and combinations of metrics) are most useful (and least obtrusive) to collect that will yield the desired patient outcomes	Choosing an RPM device by considering the patient’s needs and the type of data needed to provide patient care Determining which metrics are most appropriate to capture for a patient given their health condition and comorbidities Evaluating the business models for device purchases and maintenance
	How frequent?	Determining best practices for the frequency of RPM data collection while considering the patient’s health condition and likelihood of adhering to the schedule	Reviewing evidence-based practices to determine the frequency of RPM data collection for clinical care pathways
**Data transmission and storage**			
	How to transmit data?	Identifying systematic bias or limitations in transmitting RPM data due to lack of access to broadband, technology competence, or ability to physically visit a clinician office	Determining the data transmission approach most feasible for the patient Providing connectivity through the device (direct cell network transmission) versus leveraging patient networks and device apps
	How to secure data?	Determining means to limit the vulnerability of RPM data when transmitted and stored	Informing patients of the security, privacy, and risks of their RPM data during transmission and storage of data
	How interoperable?	Creating robust methods to maintain patient privacy when sharing RPM data across health care information systems Determining the appropriate level of granularity of data to share (raw vs summary)	Leveraging interoperability standards to ensure RPM data can be shared across systems (eg, from a device’s proprietary system to an electronic health record)
**Data analysis**			
	What algorithm?	Examining the advantages and disadvantages associated with different forms of algorithms and rules (ie, static vs dynamic) used to analyze RPM data	Understanding the implications associated with the manner in which RPM data are analyzed
	Which co-morbidities?	Identifying the interactions between comorbidities affect clinical care pathways when using RPM interventions	Determining which comorbidities to control for or consider when implementing RPM for a patient population
	What is the potential for bias?	Benchmarking and validating algorithms to detect and mitigate bias	Considering how biases within the patient’s RPM data (or across multiple patients’ RPM data) could influence the findings or recommendations
**Information presentation**			
	How to make information accessible?	Evaluating the outcomes of patients when RPM information is embedded within electronic health record systems	Ensuring the RPM information is part of the clinician’s workflow
	How to share information for decision-making?	Determining how to present information effectively to improve decision-making while avoiding cognitive overload Examining methods of presentation that encourage clinicians to use the results to augment (rather than replace) human judgment	Designing information presentation to avoid alert fatigue Developing alternative strategies, such as digital nudges, to encourage clinicians to apply RPM data in patient care decisions
	How to present information for decision-making?	Identifying factors (eg, trust, transparency, and usability) related to RPM information that affect clinician’s decision-making	Exploring presentation methods to enable effective use of RPM information for decision-making

## Abbreviations

CDS: clinical decision support
IT: information technology
RPM: remote patient monitoring
